# Gender-Specific Manifestations and Diagnostic Challenges of Autism Spectrum Disorder in Women: A Focused Study

**DOI:** 10.1192/j.eurpsy.2025.842

**Published:** 2025-08-26

**Authors:** B. Mihaela, N. Anatol, J. Dorin, V. Oprea

**Affiliations:** 1 Department of Mental Health, Medical Psychology, and Psychotherapy, Nicolae Testemițanu State University of Medicine and Pharmacy of the Republic of Moldova, Chişinǎu; 2 Department of Mental Health, Medical Psychology, and Psychotherapy, Nicolae Testemițanu State University of Medicine and Pharmacy of the Republic of Moldova, Chişinau, Moldova, Republic of

## Abstract

**Introduction:**

Autism Spectrum Disorder (ASD) is often underdiagnosed in women due to gender-specific manifestations and the use of diagnostic criteria primarily based on male presentations. This underdiagnosis can lead to delayed or inadequate support and interventions for women with ASD. There is a growing recognition of the need for gender-sensitive diagnostic approaches to better identify and support women on the spectrum.

**Objectives:**

This study aims to explore the unique manifestations of ASD in women, identify key diagnostic challenges, and propose recommendations for refining diagnostic criteria to improve accuracy and timeliness of diagnosis in female populations.

**Methods:**

A mixed-methods approach was employed, combining quantitative data from standardized ASD diagnostic tools (e.g., ADOS-2, ADI-R) and qualitative data from in-depth interviews with 50 women diagnosed with ASD. Participants were recruited from clinical settings and ASD support groups. Data were analyzed using thematic analysis to identify gender-specific behavioral patterns and diagnostic challenges, while statistical analysis compared symptom presentation between male and female groups.

**Results:**

Women with ASD exhibited distinct behavioral patterns, such as enhanced social masking abilities, higher levels of camouflaging, and differences in special interests compared to men. Table 1 highlights the frequency of common ASD symptoms in women vs. men, demonstrating significant differences in social communication (p < 0.05) and repetitive behaviors (p < 0.01). Table 2 illustrates the discrepancy in diagnostic ages between genders, with women receiving a diagnosis on average 5 years later than men. Table 3 outlines the diagnostic tools used and their respective sensitivity rates for female ASD presentation. Figure 1 visually represents the comparative analysis of symptom profiles by gender, showing a higher prevalence of internalizing symptoms in women.

**Conclusions:**

The study confirms that ASD in women often presents differently, leading to significant diagnostic delays and underdiagnosis. Gender-specific manifestations, such as social masking and camouflaging, challenge the current diagnostic criteria, which are largely based on male-centric data. To improve the diagnosis and care of women with ASD, it is crucial to adapt existing diagnostic tools to account for these gender differences and develop new guidelines that reflect a broader spectrum of presentations.

**Disclosure of Interest:**

None Declared

